# Correction to “Single‐cell RNA sequencing of pediatric Hodgkin lymphoma to study the inhibition of T cell subtypes”

**DOI:** 10.1002/hem3.70023

**Published:** 2024-10-07

de Kanter J, Steemers A, González D, et al. Single‐cell RNA sequencing of pediatric Hodgkin lymphoma to study the inhibition of T cell subtypes. *HemaSphere*. 2024;8:e149.

In the author listing of the manuscript, the first name of an author was incorrectly listed as Daniel Montiel Gonzalez. The correct name is Diego Montiel González.

The original publication has been corrected. We apologize for this error.

